# Artificial Intelligence and Machine Learning in Optical Fiber Sensors: A Review

**DOI:** 10.3390/s25247442

**Published:** 2025-12-07

**Authors:** Lidan Cao, Sabrina Abedin, Guoqiang Cui, Xingwei Wang

**Affiliations:** Electrical and Computer Engineering Department, University of Massachusetts Lowell, Lowell, MA 01854, USA; lidan_cao@student.uml.edu (L.C.);

**Keywords:** optical fiber sensors, artificial intelligence, machine learning, neural networks

## Abstract

The integration of artificial intelligence (AI) with optical fiber sensing (OFS) is transforming the capabilities of modern sensing systems, enabling smarter, more adaptive, and higher-performance solutions across diverse applications. This paper presents a comprehensive review of AI-enhanced OFS technologies, encompassing both localized sensors such as fiber Bragg gratings (FBG), Fabry–Perot (FP) interferometers, and Mach–Zehnder interferometers (MZI), and distributed sensing systems based on Rayleigh, Brillouin, and Raman scattering. A wide range of AI algorithms are discussed, including supervised learning, unsupervised learning, reinforcement learning, and deep neural architectures. The applications of AI in OFS were discussed. AI has been employed to enhance sensor design, optimize interrogation systems, and adaptively tune configurations, as well as to interpret complex sensor outputs for tasks like denoising, classification, event detection, and failure forecasting.

## 1. Introduction

Sensing technologies play an important role in modern society, enabling a wide range of applications across diverse sectors such as healthcare, manufacturing, transportation, energy, agriculture, and consumer electronics. As microelectronics, wireless communication, and data processing technologies continue to advance, sensors have evolved to become smaller, more intelligent, energy-efficient, and capable of real-time monitoring and seamless integration into complex systems such as the Internet of Things (IoT) [1]. To meet the specific requirements of different application scenarios, various types of sensing technologies have been developed, including electromagnetic sensors, piezoelectric sensors, optical sensors, and electrochemical sensors [2,3]. Among these, optical fiber sensing (OFS) technology, which leverages the modulation of light to detect changes in physical, chemical, or biological parameters, has garnered significant attention. This is largely due to its unique advantages, such as its all-glass structure, compact and lightweight form factor, immunity to electromagnetic interference, and robustness in harsh and remote environments [4]. Additionally, OFS systems can provide distributed and multiplexed sensing over long distances, making them ideal for applications in structural health monitoring, oil and gas pipelines, aerospace, and biomedical diagnostics.

OFS first emerged in the 1960s with the development of the laser. Early efforts of OFS started in 1970s and focused on intensity-based sensing, leveraging light attenuation to detect changes in environmental conditions [5] and has since evolved into a critical tool across a wide range of industrial applications. The fundamental principle of OFS involves the modulation of light properties such as intensity, phase, polarization, or wavelength within an optical fiber in response to external physical, chemical, or biological stimuli [6]. The inherent advantages of OFS include immunity to electromagnetic interference, high sensitivity, lightweight and compact form factors, and the capability for distributed sensing over extensive distances [7]. In recent years, OFS technologies have seen rapid growth, with applications ranging from structural health monitoring and seismic activity detection to biomedical diagnostics and environmental sensing. For example, distributed acoustic sensing (DAS) systems can transform conventional optical fiber cables into dense arrays of acoustic sensors, allowing real-time monitoring of pipelines, transportation systems, and subsurface geophysical events [8]. However, challenges such as cross-sensitivity to multiple physical parameters, signal attenuation, and the difficulty of real-time interpretation of large-scale sensing data continue to hinder broader deployment. To address these issues, researchers have focused on improving both the physical design of sensing systems and the intelligence of data processing tools [9]. Another promising direction has been the integration of artificial intelligence (AI) and machine learning (ML) algorithms into OFS systems. These approaches are increasingly used to tackle signal complexity, extract meaningful patterns, and enable automated decision-making. The convergence of AI and OFS improves the efficiency and reliability of sensing systems and lays the foundation for intelligent, adaptive sensor networks that can respond dynamically to complex environments and emerging demands [10,11,12].

Artificial Intelligence (AI) conceptual origins can be traced back to the mid-20th century, with milestones like the Turing Test and early symbolic reasoning systems. Over the decades, AI has evolved through several waves, from rule-based expert systems in the 1970s–1980s to the advent of machine learning (ML) in the 1990s and the deep learning revolution in the 2010s. Modern AI technologies are broadly categorized into supervised learning, where algorithms learn from labeled data, and unsupervised learning, which identifies patterns in unlabeled datasets. Other paradigms include reinforcement learning, semi-supervised learning, and self-supervised learning. Neural networks (NNs), as a foundational architecture, are used across these paradigms and can be trained in both supervised and unsupervised settings [13,14]. In recent years, AI has achieved unprecedented progress across domains. Convolutional Neural Networks (CNNs) have revolutionized image and pattern recognition, while Transformer-based models like Bidirectional Encoder Representations from Transformers (BERT) and Generative Pre-trained Transformer (GPT) have redefined natural language processing. In real-time systems, advances in lightweight architectures and on-device AI have expanded deployment in embedded and edge environments. Meanwhile, learning paradigms such as unsupervised and self-supervised learning, often implemented using neural networks, are gaining popularity because their reduced reliance on large labeled datasets supports the advancement of sensor technologies [15,16,17,18,19,20,21,22].

In recent years, AI techniques have been increasingly integrated with OFS systems for system-level optimization. In this paper, we classify the applications of AI in OFS into two distinct categories based on their purpose: AI for OFS system optimization, and AI-driven data interpretation. The first category focuses on enhancing the performance of the OFS system itself. This includes improving the signal-to-noise ratio (SNR) through deep learning-based denoising models, compensating for cross-sensitivity effects such as temperature–strain coupling using data-driven decoupling algorithms, accelerating signal processing, and enabling efficient data compression and transmission to support large-scale or real-time monitoring deployments. The second category involves AI-driven interpretation of the sensing data. Here, common techniques such as CNNs and support vector machines (SVMs) are widely employed for event classification and recognition across applications like wearable sensing and structural health monitoring. Another major application area is prediction and failure forecasting, where both supervised and unsupervised learning, including various NN architectures, have shown promise in fields such as structural health monitoring and biomedical sensing. Furthermore, AI has also been utilized for reconstructing spatial or spectral distributions in sensing systems. These advancements highlight the growing synergy between AI and OFS, paving the way for more intelligent, adaptive, and automated sensing systems.

In this paper, we first introduced the fundamental principles of OFS and established a clear classification framework encompassing point-based and distributed sensing techniques. This included an overview of the operating mechanisms of fiber Bragg grating sensors, interferometric sensors, and distributed sensing methods based on Rayleigh, Brillouin, and Raman scattering. Then presented the background of commonly used AI methodologies relevant to OFS, organized into supervised learning, unsupervised learning, reinforcement learning, and neural network-based approaches, with a focus on their core principles and representative sensing applications. Section 3 and Section 4 provided a comprehensive review of recent AI-enhanced OFS research, structured along two major application directions: (1) AI for system-level optimization and (2) AI for data-interpretation enhancement. Across these studies, we compared algorithmic performance, highlighted implementation strategies, and summarized improvements achieved in sensing accuracy, robustness, and efficiency. Finally, the concluding section synthesized the main insights, identified persistent challenges in integrating AI with OFS, and outlined future research directions essential for advancing next-generation intelligent OFS systems.

## 2. Background

### 2.1. Introduction to Optical Fiber Sensors (OFS) and Their Limitations

Properties of optical structures to measure various physical, chemical, and environmental parameters with exceptional sensitivity, immunity to electromagnetic interference, and the capacity for long-distance monitoring. These attributes make OFS a powerful choice for applications requiring high precision and reliability. Broadly, optical fiber sensors can be categorized by their sensing topology or configuration into point-based and distributed sensors. point-based sensors measure at a specific point or discrete section along the fiber. They typically involve a deliberately engineered fiber element that responds to environmental changes at that location. Common examples include Fiber Bragg Grating (FBG) sensors, various fiber interferometers including Fabry–Perot (FP), Mach-Zehnder Interferometers (MZI), and optical fiber surface plasmon resonance (SPR) sensors. In an FBG, for instance, a periodic modulation of the fiber’s core refractive index reflects a narrow wavelength which shifts in response to strain or temperature, allowing highly localized and precise measurements. Fiber interferometric sensors rely on phase changes between two light paths to sense strain, pressure, or other perturbations at a point. These point sensors often utilize standard single-mode fiber, but may also integrate sections of multimode or specialty fibers to enhance sensitivity or create the desired interference effects. They offer high sensitivity and fast response at specific locations, but by themselves cover only discrete points.

With advancements in sensing technologies, distributed optical fiber sensors (DOFS) has emerged as a transformative approach, distinct from traditional point-based sensors, DOFS systems highly enhance spatial resolution and have gained widespread adoption in recent years across various applications. Unlike point sensors, which provide localized measurements, DOFS offers the capability for continuous, spatially distributed monitoring along the entire length of the fiber. This makes them particularly suitable for environments requiring long-distance sensing and monitoring of large-scale systems and critical infrastructures. DOFS systems enable the detection and localization of changes in parameters such as temperature, strain, and other physical phenomena with high spatial resolution. This capability is invaluable for structural health monitoring, environmental sensing, and industrial asset management applications.

DOFS operates based on the scattering of light within the fiber. Different DOFS systems are optimized for specific applications depending on the type of scattering utilized. Commonly used DOFS systems include Brillouin Optical Time Domain Analysis/Reflectometry (BOTDA/BOTDR), which rely on Brillouin scattering and are particularly suited for strain and temperature measurements over long distances with high accuracy; Optical Frequency Domain Reflectometry (OFDR) and Optical Time Domain Reflectometry (OTDR), which utilize Rayleigh scattering and are used in detecting changes in strain, temperature, or faults along the fiber with fine spatial resolution; and Distributed Temperature Sensing (DTS), which is based on Raman scattering and designed for precise temperature monitoring, often used in industrial settings like pipeline and power cable monitoring. By leveraging these scattering mechanisms, DOFS provides a comprehensive sensing solution capable of addressing the challenges posed by large-scale and dynamic monitoring environments, ensuring high reliability and granularity in data acquisition [23].

OFS technologies have continually advanced, yet several challenges and limitations impede their broader deployment in certain applications. A prominent issue is cross-sensitivity. This is caused by the fact that many fiber sensors respond to multiple parameters simultaneously. For example, an FBG’s Bragg wavelength shifts due to both strain and temperature, making it hard to isolate the effect of each without additional compensation or sensors. Such temperature–strain cross-sensitivity complicates accurate measurement. Another challenge is maintaining accuracy over long ranges. In distributed sensing, signal attenuation and noise accumulation over kilometers of fiber reduce the signal-to-noise ratio and accuracy at distant points. Achieving high spatial resolution in distributed sensors also often comes at the cost of reduced range or longer acquisition times, posing a trade-off. Interrogation complexity is a further hurdle. The raw signals from fiber sensors are often complex and require elaborate processing to extract meaningful information. For instance, a Brillouin distributed sensor might produce spectral data at each location along the fiber. Demodulating this to obtain temperature or strain values involves curve fitting or frequency estimation that can be computationally intensive. Likewise, multimode speckle sensors yield high-dimensional patterns that are not straightforward to interpret. As sensing networks scale up, the volume of data to analyze in real time becomes enormous. Human or conventional algorithmic analysis of such big data in real time is error-prone or simply infeasible. Additionally, though fiber sensors are immune to electromagnetic noise, they still suffer from optical noise, baseline drift, or variability in coupling conditions.

These limitations have spurred researchers to seek more intelligent data processing and sensor management techniques, which is where Artificial Intelligence (AI) and Machine Learning (ML) come into play. AI algorithms can learn complex patterns and relationships from data, making them well suited to handle the rich and complicated outputs of advanced fiber sensors. In recent years, there is a growing recognition that embedding AI into OFS can address several needs. AI can be trained to interpret sensor signals that are too complex for manual or classical analytical methods. For instance, in distributed acoustic sensing [24], pattern recognition algorithms such as neural networks have been used to automatically identify events [25,26,27]. Data-driven models can learn to compensate for cross-sensitivity and calibration drifts. By feeding a model training data of sensors under varying conditions, the model can predict the target measurand while accounting for interference from other factors. For example, researchers have shown that an ML model can take an FBG spectrum affected by both strain and temperature and concurrently estimate both parameters, effectively decoupling the influences [28]. This multi-parameter estimation would be difficult to achieve with a purely physics-based approach without adding extra hardware sensors. A neural network can be trained to distinguish signal from noise by learning the typical patterns of each, thereby cleaning up sensor data [29,30,31]. In fiber sensing, deep denoising autoencoders and CNN-based filters have been used to suppress noise in distributed strain sensing signals, resulting in clearer measurements without hardware changes. This is crucial for detecting small events over long distances. In addition, Machine learning models can be extremely fast in execution, enabling near-real-time processing of fiber sensor data [32,33]. Instead of performing iterative curve fitting or Fourier analysis on each sensor reading, a well-trained neural network could infer the result in a single forward computation [34,35,36].

In summary, the infusion of AI into optical fiber sensing is driven by the need to overcome traditional limitations and to unlock new capabilities. By handling complexity and volume of data, compensating for non-idealities, and automating decision-making, AI transforms passive fiber sensor networks into intelligent sensing systems capable of self-optimization and sophisticated interpretation [37,38]. The next sections provide an overview of relevant AI/ML techniques and illustrate how different algorithms align with different OFS challenges.

### 2.2. Overview of AI and ML Techniques in OFS

With the rapid advancement of AI technology, numerous algorithms have been developed to address increasingly complex problems, finding applications across a wide range of fields. These advanced techniques are each tailored to specific tasks such as classification, prediction, pattern recognition, and decision-making. Broadly, AI algorithms can be categorized into four main types: supervised learning, unsupervised learning, reinforcement learning, and deep learning. These categories encompass a wide range of algorithms and techniques, each contributing uniquely to the capabilities of modern AI. As AI continues to evolve, these algorithms are becoming more efficient and adaptable, further expanding their applicability to diverse real-world challenges.

Supervised learning is a fundamental approach in machine learning where the model is trained on a dataset consisting of labeled input-output pairs. The primary objective of supervised learning is to enable the model to make precise predictions on new, unseen data based on the patterns it has learned from the training dataset. Commonly used supervised learning algorithms include Support Vector Machines (SVM) [39,40], regression algorithms such as Linear Regression (LR) and Gaussian Process Regression (GPR) [41,42], Decision Trees (DT), and Random Forest (RF) [43]. A key advantage of supervised models is their accuracy on well-characterized tasks. On the other hand, they require labeled datasets which can be costly to obtain. In summary, supervised learning excels at tasks like event classification, fault detection, or calibration when prior examples are available.

Unsupervised learning deals with unlabeled data, aiming to discover patterns, groupings, or anomalies inherently present in the inputs. The algorithm is not told what to predict, but rather to find structure in the data. Common unsupervised techniques include clustering methods such as k-means or hierarchical clustering which group similar data points together, and dimensionality reduction methods like Principal Component Analysis (PCA), or more complex neural network-based approaches that find simpler representations of data. In the realm of fiber sensors, unsupervised learning is particularly valuable for anomaly detection. Advanced unsupervised models used in recent years include autoencoders (AEs) and generative adversarial networks (GANs), which learn the underlying data distribution and can flag data that deviates from the learned manifold. Autoencoders have been used to learn a compressed representation of fiber optic sensor signals. The strength of unsupervised learning is its ability to leverage large amounts of unlabeled sensor data to find latent structure. However, interpreting the results can be challenging, as the algorithm might find clusters or principal components that are not immediately meaningful physically. Despite that, unsupervised techniques are indispensable for exploratory analysis and for monitoring systems where one must be alerted to any deviation from normal behavior without explicitly enumerating all possibilities beforehand.

Reinforcement learning (RL) addresses sequential decision-making problems through a trial-and-error approach. Commonly used RLs are Q-learning, Deep Q Network (DQN), Policy Gradient Methods, etc. In the context of optical fiber sensing, reinforcement learning is not yet as widely applied as supervised/unsupervised learning, but it holds promise for adaptive system optimization. A challenge with RL is the large number of iterations often required for learning, which can be computationally expensive and requires a realistic training environment for the agent. As simulation tools for fiber optic systems improve, RL-based optimization still has potential for sensor networks and real-time interrogation systems.

Deep learning refers to the use of multi-layered artificial neural networks (ANNs) to model complex patterns in data. Neural networks consist of interconnected layers of artificial neurons that apply nonlinear transformations to the data, gradually extracting higher-level features. Owing to their ability to approximate highly complex, nonlinear relationships, deep neural networks have become a cornerstone of modern AI. Several specialized architectures are relevant to OFS data [44,45,46]. Multilayer perceptron (MLP) are basic ANN structures useful for regression or simple classification on sensor data. Convolutional Neural Networks (CNNs) are powerful for data with spatial or sequential structure. They automatically learn features from local data portions and have seen success in fiber sensing for analyzing spatially distributed signals or spectrograms. For instance, CNNs have been used to recognize vibration patterns in DAS or to filter out noise from Rayleigh backscatter signals, taking raw signal segments as input and outputting a classification or a cleaned signal. Recurrent Neural Networks (RNNs), and especially their gated variants like Long Short-Term Memory networks (LSTMs), are designed to handle sequential time-series data by retaining state across time steps. They are naturally suited for processing the time-dependent output of fiber sensors, such as dynamic strain measurements or acoustic signals, where temporal correlations are important. Indeed, LSTM-based models have been applied to fiber interferometer signals to improve demodulation accuracy by accounting for temporal context. Beyond these, other deep architectures like autoencoders and deep belief networks have niche applications in sensor data compression and pattern discovery. The main advantage of deep learning is its capacity to handle raw, high-dimensional data and automatically extract meaningful features, which is particularly useful for complex sensing signals. However, deep networks typically require large datasets for training and substantial computational resources, and their inner workings are often a “black box,” which can be a drawback in safety-critical sensing applications that demand interpretability. Despite these challenges, the past few years have seen an explosion of deep learning applications in optical sensing, riding on the general AI boom and improved computing capabilities.

In summary, the applications of AI/ML in OFS range from simple, interpretable models to sophisticated deep neural networks. Table 1 summarizes these categories and example algorithms. The choice of algorithm depends on the problem at hand and the data available. The following sections focus on the applications of artificial intelligence in OFS, which can be broadly categorized into two areas: (1) enhancing the performance of the sensing system itself, and (2) improving the interpretation and analysis of sensor data. For each area, recent studies will be reviewed in detail, with particular attention to the AI/ML algorithms employed. Comparative analysis of their performance and outcomes will also be presented.

## 3. AI in OFS: Sensing System Optimization

OFS system benefits greatly from recent AI and ML techniques, which have been leveraged to enhance signal quality and system performance in various ways. Key areas of improvement include signal-to-noise ratio (SNR) enhancement, cross-sensitivity compensation, accelerated signal processing, and efficient data compression and transmission. Boosting SNR allows longer sensing range and finer resolution, while compensating cross-sensitivity enables accurate multi-parameter measurements. Likewise, faster processing and data reduction are crucial for real-time monitoring and handling the massive data streams produced by distributed sensors.

### 3.1. SNR Enhancement Using AI

OFS system often suffers from noise due to scattering, attenuation, and external perturbations, which can obscure the measurand. Traditionally, improving SNR involves hardware solutions or signal processing like filtering and averaging. However, conventional methods struggle when the signal is extremely weak or the noise spectral characteristics overlap with the signal. AI offers a data-driven approach to denoising, learning the statistical or physical relationship between noisy outputs and true signals. In effect, a trained model can act as an intelligent filter, extracting weak signals that would otherwise be lost in noise. The AI does this by being trained on examples of the sensor output under various noise conditions, learning to map the noisy input to the true value.

A variety of neural network architectures have been explored for SNR enhancement in fiber sensing. For instance, Feng et al., 2022 [47] applied a trained convolutional neural network to map the captured speckle pattern to an input spectrum based on MMF sensor. By learning this mapping, the AI essentially decodes the wavelength-encoded speckle and ignores modal noise. The model learns to filter out random speckle fluctuations and camera noise, yielding a clearer output spectrum. This improves the effective SNR of the spectral reconstruction. In experiments, they achieve a spectral resolution on the order of 0.1pm and recovery of thousands of spectral lines and report large reductions in reconstruction error. In practical terms, the AI decoder delivers higher spectral precision and a higher output SNR compared to a non-ML approach. Nguyen et al., 2021 [48] trained a deep neural network to predict temperature from the noisy spectrum. They also compared a multilayer perceptron vs. a 1D convolutional network. The chosen AI model is a fully connected DNN (MLP), which outperforms a CNN for this 1D spectral data. The network learns to associate subtle spectral shifts with temperature even when white noise perturbations are present, effectively treating the noise as a distractor. The result is a dramatic SNR improvement: while traditional methods like peak detection or Fourier phase-shift only recovered the correct temperature 15–40% of the time under heavy noise, the DNN achieved near-100% accuracy.

Beyond these examples, many other studies apply AI to improve SNR in OFS. Convolutional denoising autoencoders have been trained on noisy Brillouin gain spectra to produce cleaned signals, cutting frequency uncertainty in half. CNNs and Forward Neural Networks (FNNs) have been used to denoise distributed acoustic or temperature data, yielding significantly lower noise floors. Lalam et al., 2024 [49] showed that DNN-based denoising reduced the measured Brillouin frequency shift uncertainty from ~3 MHz to ~1.2 MHz. Likewise, Lapins et al., 2024 [50] developed a Noise2Noise deep learning approach for DAS that learned to map raw noisy signals to clean ones without needing noise-free labels. This method outperformed conventional band-pass filters, greatly increasing the detectability of small vibrations.

Across these approaches, FNNs, CNNs, and AE are the predominant algorithms for fiber optic denoising. Simpler MLPs have been shown effective for one-dimensional spectra, while CNNs excel at exploiting local spectral patterns in distributed or image-like data. LSTMs are also used when temporal noise correlations are important. The results universally show improved SNR or reduced measurement error. For example, in a Brillouin sensor, applying a DNN cut the measurement uncertainty by more than half. Many works report that the noise floor of the sensor system is significantly lowered, enabling detection of smaller signal changes. Essentially, the AI learns from training examples to preserve the true physical signal while suppressing random fluctuations [51,52].

The use of AI for SNR enhancement in OFS has proven to be a powerful augmentation to physical sensing. Importantly, these methods operate in software, avoiding the need for expensive hardware modifications or extreme noise-shielding measures. Once trained, the model inference is fast, allowing real-time denoising in field deployments. A potential challenge is the need for representative training data. The model must see examples of the signal under the noise conditions of interest. Nonetheless, studies have shown that even simulated data or semi-supervised approaches can be used to train effective denoisers. Overall, AI-driven denoising has dramatically improved the reliability of fiber sensors, extending their usable range and sensitivity by extracting signals that would otherwise be overwhelmed by noise.

### 3.2. Cross-Sensitivity Compensation with AI

A longstanding challenge in optical fiber sensing is cross-sensitivity, where a single sensor responds to multiple physical parameters. In conventional systems, additional reference sensors or calibration matrices are required to separate these effects. However, these approaches may be inaccurate when the responses are nonlinear or more than two parameters are coupled. AI provides a flexible solution by learning the direct mapping from the sensor’s raw signal to the individual quantities of interest. Essentially, a model can be trained on multi-parameter data so that it inverts the cross-influences and outputs independent measurements of each target parameter.

Many researchers have applied neural networks to tackle cross-sensitivity in fiber sensors. ANNs were used as early as 2007 by Sun et al. [53] to simultaneously measure bending curvature and temperature with a pair of long-period fiber gratings (LPFGs). By feeding the two LPFG signals into an ANN, they could predict curvature and temperature with root-mean-square errors of just 0.0072 m^−1^ and 0.19 °C, respectively. More recently, multi-layer perceptrons and radial-basis function nets have been applied to FBG sensor arrays to separate influences. For instance, Saha et al., 2025 [54] developed a machine-learning-augmented FBG sensing system for structural health monitoring that used four collocated FBGs and an ML model to discriminate strain changes from temperature drift. The ML model effectively isolated mechanical strain effects even when temperature fluctuated, addressing the cross-sensitivity without needing a physical reference sensor. In another work, Zhu et al., 2022 [55] created a single-mode fiber—hollow-core fiber—single-mode fiber (SMF–HCF–SMF) heterostructure, essentially an inline interferometer with a capillary section, to measure bending. Its transmission spectrum is rich but nontrivial to interpret by conventional peak-tracking. To deal with the cross-sensitive problem, they trained a shallow ANN with one hidden layer with 5 neurons using the raw transmission spectrum as input to predict the bending curvature, including up/down direction. By learning directly from the entire spectrum, the ANN inherently compensates for cross-coupling between bending magnitude and orientation. The ML approach dramatically outperformed the classic dip-tracking method, showing about 94% of the ANN’s predictions had curvature error under 0.07 m^−1^, versus only 52% for the conventional method. The network could reliably measure curvatures from 0 up to 13.67 m^−1^ and even determine bending direction, a new capability not achieved by earlier methods. Karapanagiotis et al., 2023 [56] applied ML to a BOFDA system on standard SMF-28 fiber. They coiled 30 m of fiber in a tensioning setup inside a climate chamber and varied temperature from 20 to 40 °C and strain from 0–1380 µε. High-SNR BOFDA scans yielded spectra with four resolved Brillouin peaks at each location. They extracted the four peak frequency shifts as features for regression. To compensate for temperature and strain cross-sensitivity, they tested simple frequentist models like linear regression and Bayesian methods, finding that GPR gave the best performance. The GPR model mapped the Brillouin peak shifts to temperature and strain simultaneously, effectively decoupling the two effects. Using leave-one-out validation, the ML model achieved temperature and strain errors of about 2 °C and 45 µε, respectively, essentially eliminating the conventional crosstalk between these parameters.

Beyond these examples, ML has also been implemented in long-period grating (LPG) sensors to separate refractive index changes from temperature, in FP interferometers to distinguish pressure vs. temperature effects, and in hybrid distributed sensors to distinguish strain from temperature along the fiber. In each case, the algorithm is trained on calibration data where the multiple parameters were varied, enabling it to later solve the inverse problem of retrieving individual measurands from a composite signal.

In summary, supervised learning is the dominant approach for cross-sensitivity compensation. Common algorithms include feed-forward ANNs and sometimes fuzzy inference systems or Gaussian processes when uncertainty quantification is desired. These models typically take either the full sensor spectrum or a set of extracted spectral features as input, and output the estimated values of each measurand. The success metrics reported are significantly improved accuracy of each parameter measurement without needing separate sensors. For example, the ANN in the bending sensor provided a new capability and a large error reduction compared to spectral peak tracking. The GPR in the Brillouin sensing example essentially eliminated the temperature–strain cross talk, yielding accuracy comparable to having two separate calibrated sensors. The flexibility of ML means it can adapt to nonlinear and unexpected coupling effects that are hard to model analytically.

AI-based cross-sensitivity compensation allows fiber sensors to become true multi-parameter devices without sacrificing accuracy in any channel. It simplifies sensor networks and improves robustness. A challenge is that a robust training dataset covering the full range of each parameter is required; collecting such data can be time-consuming. There is also a risk that the ML model might not generalize beyond the conditions it was trained on. Researchers address this via regularization and by including a wide range of conditions in training, or retraining the model in situ if needed. Overall, the literature demonstrates that data-driven decoupling is highly effective from point sensors like FBGs and LPGs up to distributed sensors, where AI can separate. This capability is a key enabler for deploying OFS in environments where multiple stimuli act on the sensor simultaneously.

### 3.3. Accelerating Signal Processing with AI

Many optical fiber sensors, especially interferometric types, require intensive signal processing to extract the measurand. For instance, demodulating an interferometer often involves peak-finding or phase-unwrapping algorithms that can be iterative and slow. Similarly, interrogating a high-density FBG array or distributed sensor may demand real-time spectral analysis that pushes conventional processing to its limits. These bottlenecks can limit the sensor’s sampling rate or introduce latency. AI/ML models offer a solution by learning a direct mapping from raw sensor signals to the desired output, effectively skipping computationally heavy steps. By replacing algorithmic demodulation with a trained model’s prediction, one can achieve near-instantaneous readout. A clear example is the work by Zhu et al., 2023 [57]. They applied ML to demodulate a Vernier-effect dual-interferometer sensor. Their model takes the combined spectrum and directly outputs the measured quantity, enabling fast, direct, and reliable readout. This eliminates the need for conventional peak-tracking or fringe-fitting methods, drastically accelerating the processing. In a complementary study, Xue et al., 2023 [58], developed a fiber Michelson interferometer for chemical concentration sensing and used an LSTM network to demodulate its spectrum. The LSTM model achieved 97.5% accuracy, significantly outperforming traditional peak-wavelength tracking by avoiding errors caused by dip selection, sampling rate, and noise. Other recent efforts have similarly applied deep neural networks to speed up fiber sensor interrogation, demonstrating accelerated signal processing across diverse OFS platforms. Other researchers have reported similar successes. Deep neural networks have been used as virtual spectrometers for FBG sensor arrays, identifying Bragg wavelengths from raw detector signals in milliseconds. A dilated CNN approach by Li et al., 2021 [59] could demodulate overlapping FBG signals from two sensors in only ~15 ms, enabling real-time monitoring even when spectra overlap. More complex architectures like Transformer networks have also been employed. These ML models perform the equivalent of spectral decomposition or curve-fitting much faster by leveraging their training on many examples.

The common thread in these approaches is using supervised learning regression to approximate the sensor’s transfer function. Algorithms range from simple FFN to 1D-CNNs and sequence models like LSTM for sensors that produce sequential signals. The speedups reported are substantial. The Vernier sensor ML demodulator enabled essentially instantaneous measurements where a traditional algorithm might take seconds. The LSTM demodulator for the Michelson interferometer not only improved accuracy but allowed high-rate sampling by processing data in real time. In high-density FBG networks, using cloud-deployed deep learning has enabled near-real-time inference even as the number of sensors scales up, something that would be very challenging with classical peak-fitting for each sensor. The accuracy of these ML demodulators is typically on par with or better than the conventional method they replace.

Fast AI-based processing is unlocking new possibilities in OFS. For example, enabling real-time distributed acoustic sensing for intrusion or seismic monitoring, where the ML can classify events on the fly. By drastically reducing latency, AI demodulation allows fiber sensors to be used in active feedback or high-speed control systems. A consideration is that the ML model must be trained across the full range of sensor responses. If the system goes outside this range, the model might extrapolate unpredictably. In practice, many works demonstrate the models are quite robust within their design range, and they can be retrained or updated if the sensor response drifts over time. Another consideration is computational load. A large neural net could itself be slow, but most cited approaches use relatively compact networks that run on standard CPUs or small edge devices in microseconds to milliseconds. Overall, the trend is that AI is bypassing classical computational bottlenecks in OFS.

### 3.4. Data Compression and Efficient Transmission

Distributed and long-term fiber sensor deployments generate massive data streams. Transmitting or storing all raw data is often infeasible. Conventionally, this is handled by downsampling, simple threshold-based event triggers, or lossless compression algorithms, but these either risk losing important information or still result in large data volumes. AI techniques offer smarter ways to perform feature extraction and compression, essentially picking out the important information from the raw data so that only the concise information needs to be transmitted or stored. Wang et al., 2023 [60], demonstrated this in a fiber-based ballistocardiograph (BCG) system. Long BCG waveforms encode heart rate (HR) and respiration rate (RR); their approach used an improved empirical mode decomposition (VEMD) to decompose the signal and an LSTM-based model to predict HR and RR from the components. This deep-learning pipeline achieved an R-squared of 0.9946 on test data, effectively compressing lengthy optical waveforms into just two vital-sign values. In another example, Peng et al., 2020 [61], applied AI to a phase-sensitive OTDR (φ-OTDR) pipeline sensor for intrusion detection and corrosion monitoring. Their neural network processed continuous DAS data in real time, classifying external impacts with over 85% accuracy and identifying pipe defects with over 94% accuracy (71% unsupervised). In this case, the raw DAS time series was reduced to discrete alarms and condition flags. Compared to Wang’s single-fiber HR/RR regression, Peng’s system used a long fiber array and trained classifiers, yet both achieved dramatic data reduction. In each case, the ML model compressed the sensor signal enabling the efficient transmission of only essential information. AE have also been directly applied for compression. For instance a recurrent autoencoder (RAE) was proposed by Wang et al., 2024 [62] for DAS seismic signals. Their deep autoencoder, augmented with LSTM layers, achieved compression ratios up to 512:1 with only minor loss in fidelity, reconstructing the signal with >40 dB SNR at 8× compression and still ~24 dB at 64× compression. This far outperforms standard compression on the same data. Other studies have implemented neural encoders for fiber networks, where a trained model at the node compresses sensor readings and the decoder at the server reconstructs the signals or directly outputs decisions.

Key AI techniques for data reduction in OFS include feature-extracting NNs, such as classifiers, regressors, and autoencoders. In all cases, the improvement is measured by how much the data volume is reduced versus how much useful information is preserved. The BCG example compresses minutes of waveform into two numbers with essentially no loss of diagnostic content. The φ-OTDR example reduces a continuous data stream into occasional labeled events, which is a drastic compression enabling 24/7 monitoring with minimal bandwidth. The RAE example shows >500× data size reduction for DAS signals while still allowing reconstruction with SNR high enough for practical use. These approaches often report near-lossless reconstruction or detection. Compared to traditional compression, the ML approaches are far more efficient because they are tailored to the specific patterns in fiber sensor signals. AI-driven compression aligns the data output with what the end user or system actually needs rather than sending raw readings. This reduces the burden on data storage and transmission in OFS networks, which is critical for remote or long-term deployments.

### 3.5. Discussion of AI in OFS for System Optimization

Across the areas of system-level optimization including SNR, cross-sensitivity, speed, and compression, it is evident that AI techniques are transforming how OFS operates. A unifying theme is that machine learning enables a shift from hardware or manual solutions to smart, adaptive software solutions. Rather than physically redesigning a sensor to improve SNR or adding extra reference fibers for cross-sensitivity compensation, deploying a learning algorithm can achieve similar or superior outcomes by leveraging patterns in the data. This greatly enhances the versatility of OFS. The same raw sensor can be re-purposed for different measurands or operating conditions by switching the model it runs with. Another notable point is the inter-connected benefit of these improvements. For example, by denoising and boosting SNR, AI not only makes the readings more accurate but often also allows faster processing and better compression. Conversely, by compressing data and focusing on key features, AI can also effectively improve SNR in the sense of filtering out noise. In practice, studies often find that their ML solution simultaneously addresses multiple challenges. This holistic improvement is very promising for real-world applications. Reliability and validation remain important considerations. Introducing AI into a sensing system means it must be rigorously validated under diverse conditions to ensure the model does not introduce biases or errors. The explainability of some models can be limited. While a simple regression might be easy to understand, a deep neural network acting on a spectrum is essentially a black box. Researchers are exploring physics-informed neural networks and other hybrid approaches to retain interpretability. For example, incorporating known physical relationships into the model architecture can ensure it behaves reasonably outside the training data range.

In summary, AI methods for OFS system optimization have shown significant performance gains in recent literature. Table 2 below compares a selection of representative studies and their outcomes. These data-driven techniques allow fiber sensors to reach new levels of sensitivity, accuracy, speed, and efficiency. The convergence of photonics and AI thus appears to be a key driving force in the next generation of optical fiber sensing systems, enabling smarter sensing networks that can adapt and learn from their environments.

## 4. AI in OFS: Data Interpretation Enhancement

The raw data produced by OFS often contains complex patterns and massive volumes of information, posing challenges for traditional analysis. AI techniques have emerged as powerful tools to enhance the interpretation of sensor data, enabling more accurate event identification and predictive insights. In particular, two key application areas have gained prominence in recent years: event classification, where AI algorithms discern and label different disturbances or phenomena from optical fiber signals, and prediction and reconstruction tasks, where AI models infer future states or reconstruct underlying physical parameters from sensor data.

### 4.1. Event Classification

Early approaches to event analysis in OFS relied on heuristic thresholding or simple signal processing, which struggled with noisy, high-dimensional data. By around 2018, researchers began applying ML to recognize patterns in OFS signals. It offered initial improvements over manual analysis, but they required extensive feature engineering and had limited ability to cope with the variability in real-world signals. Today, the state-of-the-art is defined by deep learning models that automatically learn discriminative features from raw fiber sensor data, achieving near-real-time recognition of diverse event types with high accuracy.

A representative example of classical ML in OFS is the work by Fouda et al., 2021 [66], who analyzed vibration signals from submarine telecommunication cables for security monitoring. They combined time–frequency feature extraction with an SVM classifier to identify malicious disturbances on multiple buried cables. Their SVM-based system achieved over 96% accuracy in distinguishing three typical event classes, demonstrating the feasibility of pattern recognition for fiber optic sensors. However, such approaches reach a performance plateau when faced with more complex or noisy data, as they rely on predefined features and linear decision boundaries. Deep learning approaches have since pushed this frontier. Wu et al., 2019 [67] proposed a one-dimensional CNN combined with SVM for in situ pipeline monitoring using DAS. Their CNN was trained on vibration data from a φ-OTDR fiber running along a pipeline, and it learned to automatically extract salient features for event classification. The model distinguished common pipeline events with 97–98% accuracy and could output results within 1 s of data acquisition. Liu et al., 2023 [68] used an actively heated fiber Bragg grating (AH-FBG) array buried in soil to monitor moisture. They measured the temperature rise in the fiber under three surface covers (bare soil, grass, biochar-soil) and found that cover conditions affected the thermal response. An ANN was trained on the FBG temperature data to predict soil water content under these varying covers. The ANN significantly improved moisture estimation accuracy compared to simple calibration, effectively learning to compensate for cover-induced variations. Zhuang et al., 2021 [69] embedded a single FBG in a smart helmet to capture head impacts. Blunt impacts cause transient strain signals in the helmet, and each impact with a different magnitude, direction, and location produces a unique “fingerprint” in the FBG reflection. They created a training set of impact signals using known strikes, then trained multiple ML models to predict the impact parameters. They tested SVM, multilayer perceptron (ANN), and RF, as well as boosted variants like “SVM” and “IBK.” These classifiers were used to map the FBG signal to impact magnitude and direction. The best models achieved very high accuracy and predictions of impact magnitude and direction had an R^2^ ≈ 0.90 on unseen data. In effect, the AI classified each impact event by estimating its strength and angle. This system could thus recognize and label concussive events in real time, with AI improving over what a simple threshold sensor could do. Naku et al., 2023 [70] combined an array of three fiber-tip FP sensors (intrinsic, hydrophobic-coated, and hydrophilic-coated ends) to identify volatile organic liquids (VOLs). In experiments, a droplet of each liquid was placed on the fiber tip and its evaporation was monitored as a time-series signal. The different coatings changed the evaporation dynamics, yielding richer data. They converted each transient response into a time–frequency image via continuous wavelet transforms, then fed these images into a CNN for classification. CNN was trained to label which liquid out of 11 types produced the signal. Using the diverse sensor array and the wavelet–CNN pipeline, they achieved nearly 100% classification accuracy in many cases. Recurrent neural networks (RNNs) have been applied to capture temporal patterns in OFS data, especially for events with distinctive time signatures.

A notable example is the RNN-DAS model by Fernández-Carabantes et al., 2025 [71]. They targeted volcano-induced seismic events recorded by a submarine DAS array during the 2021 La Palma eruption. Using an LSTM, their system analyzed the distributed strain signals over time to detect and classify volcano-tectonic (VT) events. Trained on an extensive dataset (~2000 labeled events), RNN-DAS achieved about 97% classification accuracy in correctly identifying VT event waveforms. This RNN-based approach is well suited to scenarios like seismic monitoring where the temporal evolution of the signal is crucial for distinguishing event types. Beyond CNNs and RNNs, advanced architectures incorporating attention mechanisms and multi-scale feature extraction are emerging. He et al., 2022 [72] introduced a dual-stage recognition network for optical fiber perimeter security, in which a first stage quickly flags potential disturbances and a second stage with a deeper CNN verifies the event type. Such two-stage systems can reduce false alarms by combining speed and precision.

These studies collectively demonstrate that AI is highly effective for event classification in OFS. OFS generates enormous data streams with subtle signal patterns. AI methods excel at handling such high-dimensional data, automatically discovering features that human-designed filters might miss [27]. This leads to superior accuracy in distinguishing events, as seen by multiple reports of >95% success rates. All these studies deal with multi-class classification of events like intrusion, activity, or environmental incidents recorded by fiber sensors. Notably, most algorithms are supervised, trained on labeled examples of each event class. AI models are also adaptable that they can be retrained or fine-tuned for new environments or fiber deployments. Furthermore, AI enables multi-class discrimination, which is invaluable for decision-making in security, infrastructure management, and geophysical monitoring. The main challenges are the need for labeled training data and ensuring robustness to novel conditions. Recent works address data scarcity via augmentation and improve generalization through architecture choices Overall, AI-driven classification has transformed OFS from simple detectors into intelligent sensing systems capable of understanding their environment and providing rich, actionable information.

### 4.2. Prediction and Reconstruction

Beyond classifying known events, AI is increasingly used to predict future sensor readings or reconstruct underlying physical fields from OFS data. These tasks leverage AI’s ability to model complex input-output relationships, enabling predictive maintenance, forecasting of environmental changes, and high-fidelity reconstruction of measurands that are difficult to obtain directly. For example, the combination of OFS with AI allows operators to anticipate failures or anomalies. Similarly, AI-driven reconstruction can infer a spatial distribution from indirect or noisy measurements. The state-of-the-art in this domain involves deep learning models that are trained on large datasets of sensor measurements and ground-truth outcomes, achieving accuracy beyond traditional physics-based models. The focus here is on two aspects: temporal prediction, where AI forecasts future behavior or detects trends, and spatial/physical reconstruction, where AI maps sensor signals to spatially distributed quantities or enhanced measurements.

Fan et al., 2023 [73] considered a basalt-fiber-reinforced polymer (BFRP) pipeline with an embedded FBG sensor network to monitor strain under internal pressure. A numerical finite-element model generated distributed strain patterns for various damage scenarios. The AI model was an enhanced CNN (ECNN), which was a CNN whose final softmax layer was replaced by a k-nearest-neighbor (k-NN) classifier for inference. The ECNN was trained on simulated strain-displacement fields to classify the pipeline’s deterioration state and thus predict damage initiation and location. Results showed the ECNN predictions matched experimental FBG strains with only 0.093% average error, and it achieved over 90% classification accuracy and F1 scores in detecting early-stage damage. This work showed that the hybrid CNN–kNN model effectively predicted pipeline failure states from the FBG data. Zhang et al., 2023 [74] used AI to predict fiber parameters from target performance. They considered a hollow-core anti-resonant (HC-AR) optical fiber, aiming to find geometric parameters that yield a desired confinement loss. They built a dataset linking fiber structural parameters to confinement loss. The AI solution was a tandem neural network (T-NN) that a forward prediction network, which mapped geometry to loss, and an inverse design network trained jointly. With a 6-layer CNN forward model, they attained a mean squared error of 7 × 10^−4^ and R^2^ ≈ 0.915 in predicting loss. The full T-NN achieved an even higher correspondence (R^2^ up to 0.988) between desired and predicted fiber designs in one dataset. In practice, the trained model could take a target loss spectrum and output fiber design parameters nearly matching that spectrum.

These examples illustrate a variety of OFS modalities, ML models, tasks, and metrics. Beyond these specific studies, AI and ML have been applied widely for predictive analysis in fiber sensing [75,76]. Support-vector machines have been used for pipeline leak forecasting, where vibration data from fiber sensors are classified to pre-warning events and localize leaks. More broadly, integrating FOS with AI and ML enables real-time anomaly detection and remaining-life estimation: the models can learn from historical sensor streams to forecast degradation, supporting proactive maintenance. In summary, these works demonstrate that AI-driven analysis of OFS data can effectively predict structural health outcomes, enabling early warnings and maintenance planning across infrastructure, energy, and industrial systems.

Another application of AI in OFS is reconstruction. OFS systems can suffer from insufficient, missing, or degraded data, requiring reconstruction. Deep learning can restore or enhance signals and images from limited optical data. For instance, Chen et al., 2022 [52] addressed the fading problem in a Mach–Zehnder BCG interferometer by training a modified GAN to reconstruct cardiac BCG waveforms. The GAN takes the distorted interferometric output and generates a cleaned BCG signal; on test data, the reconstructed BCG correlated 0.952 with a reference waveform. This eliminates the need for additional modulators or demodulators, simplifying the hardware. In another study, Hou et al., 2024 [51] fabricated a tapered multimode fiber that supports higher-order modes, which are sensitive to contact position and force. They then applied a multi-scale CNN with an LSTM layer to decode the sensor output into 2D position/force maps. The trained model achieved a spatial resolution of 7.6 μm and force resolution of 0.02 N over a 5.6 mm sensor length, effectively reconstructing fine-scale maps from a single fiber signal. Comparing these works, Chen’s GAN focuses on one-dimensional waveform recovery with high correlation, while Hou’s CNN + LSTM addresses spatial reconstruction in a short fiber section with sub-ten-micron accuracy. Both use deep networks to overcome the physical limits of fiber. Other recent efforts likewise leverage AI for reconstruction in fiber optics. These studies indicate a growing trend of ML-driven signal and image reconstruction in optical fiber sensing.

The above works demonstrate that AI techniques are remarkably well suited to prediction and reconstruction tasks in OFS. Many such problems are inverse or forecasting problems that are difficult to solve with physics-based modeling alone. For example, reconstructing a distributed temperature profile from a noisy Brillouin spectrum or predicting the remaining life of a structure from strain history involves complex, non-linear relationships. AI can learn these relationships directly from data, without needing an explicit physical model, or can complement physics by encoding known constraints. The results show that AI models have achieved higher accuracy and faster computation. This enhances the interpretability of OFS data in real time. Distributed fiber sensors provide a wealth of data, and AI algorithms are capable of distilling this into actionable insights. However, challenges are AI models require extensive training data that cover the range of expected conditions, and ensuring they generalize outside the training distribution is an active area of research. Some models, like those in structural health monitoring, may need periodic re-training or adaptation as the structure’s behavior changes over years. However, methods like transfer learning and continual learning are being explored to address this. In many cases, the combination of physics knowledge with data-driven learning is yielding robust solutions. constraining a network with known physical laws of heat diffusion when reconstructing a temperature field, to prevent unphysical predictions. In summary, AI has proven to be a powerful enabler for using optical fiber sensors not just for sensing the present, but also for predicting the future and revealing the unseen. It extends the reach of fiber sensing technology into proactive and high-resolution domains that were previously unattainable.

### 4.3. Discussion of AI in OFS for Data Interpretation

The two application areas reviewed both highlight how AI techniques significantly enhance the interpretation of OFS data. From this recent research, several integrative observations can be made. First, deep learning dominates recent literature, reflecting its superior performance with complex fiber sensor data. Simpler models like SVM or Generalized Linear Model (GLM) still appear, but hybrid and deep models are prevalent in achieving state-of-the-art results. Second, 79 the performance levels reported are generally very high accuracy, and reconstruction studies report substantial error reductions or very high correlation with ground truth. This indicates that AI is effectively addressing the challenges inherent in fiber optic sensor signals. Third, AI models are enabling real-time or near-real-time processing even for data-intensive distributed sensors. This is crucial for practical deployment. The adoption of strategies like two-stage inference (for efficiency) and dedicated AI accelerators means even complex models (CNNs, LSTMs) can run within the stringent time constraints of sensing systems.

Another important point is the generalization and transferability of AI solutions. Many OFS applications are trained on one specific fiber installation or one type of event. A concern is whether an AI model trained in one context works in another. Some studies have shown promising generalization, suggesting that with proper training, models can be made robust across different deployments. Nonetheless, the need for extensive labeled data in each new scenario is a bottleneck. Recent works emphasize creating open datasets for fiber sensing and using data augmentation or simulation to expand training coverage. This integrative approach of combining real and synthetic data is likely to be key in training AI for fiber sensing at scale. In terms of suitability, it is evident that AI’s ability to handle non-linear, multivariate relationships and to improve with more data naturally complements the physics of OFS, which often involves complex signal patterns.

Finally, it is worth summarizing the key advantages and remaining challenges in an integrated manner. AI unquestionably improves sensitivity and specificity. Events that were once missed or misclassified can now be correctly identified and subtle effects can be accurately extracted. This extends the usable range and fidelity of optical fiber sensors, making them competitive for applications requiring high reliability. On the challenge side, one must ensure these AI models are robust to out-of-distribution inputs. Explainability is also a consideration. In conclusion, AI has emerged as a transformative tool for enhancing OFS data interpretation, enabling both instantaneous understanding of sensor signals and forward-looking insights. The integration of AI algorithms with OFS networks is giving rise to a new generation of smart sensing systems. These systems are not only sensitive and distributed, as optical fiber sensors inherently are, but also intelligent and predictive, capable of learning from data, improving over time, and providing a much richer situational awareness.

## 5. Conclusions and Future Perspectives

In summary, the convergence of AI techniques with OFS has significantly enhanced both sensor performance and data analytics capabilities. Prior sections of this review outlined how AI-driven methods improve OFS system optimization and data interpretation. On the system side, machine learning models have been leveraged to boost signal quality and system efficiency temperature coupling, and learning-based approaches optimize interrogation speed and data compression for large-scale deployments. Concurrently, AI techniques empower richer data interpretation of optical fiber signals: classical classifiers and modern deep networks enable automated event classification and pattern recognition in applications from wearable biosensing to structural health monitoring. Additionally, neural network models have been applied for prediction and forecasting and even for reconstructing spatial/spectral distributions from raw fiber signals. Collectively, these advances illustrate how AI methodologies accelerate sensor design and calibration, uncover complex signal patterns, and ultimately yield more intelligent, adaptive optical fiber sensing systems.

However, translating these promising results from lab studies into robust real-world OFS deployments remains non-trivial. Practical deployment challenges span multiple fronts: calibration drift and sensor aging can alter a fiber sensor’s response over time, requiring AI models to adapt to slowly changing baselines; environmental variability can degrade model accuracy when conditions deviate from the training set; and domain shifts occur when an algorithm trained on one fiber type or installation is applied to a different context. In many cases, AI models that perform well on curated lab data struggle to generalize under new field conditions. Compounding these issues is the scarcity of labeled data for many specialized sensing tasks which increases the risk of overfitting and model brittleness. Moreover, fiber sensing applications often demand real-time or near-real-time processing, placing strict latency and efficiency constraints on the AI algorithms and hardware. These practical challenges must be carefully addressed to ensure AI-enhanced OFS can reliably move from proof-of-concept to field-deployed technology.

Beyond these general challenges, there are several major research bottlenecks that have been holding back broader adoption of AI in OFS like lack of standardized datasets and benchmarks, limited model generalization across fiber types and conditions, inadequate real-world validation and field testing, and limited integration with physics-based models. Addressing the above limitations will pave the way for the next generation of intelligent OFS. Based on the current trends and gaps identified, future research and development in AI-enhanced OFS may have directions: (1) Data-efficient and self-supervised learning that exploit the abundance of unlabeled sensor data to improve feature representations without large labeled datasets. (2) Edge computing and real-time AI deployment. (3) Open-source datasets and collaborative testbeds.

In conclusion, recent demonstrations have proven the potential for smarter, more adaptive OFS that can learn, adapt, and predict beyond the capabilities of conventional systems. To fully realize this vision, researchers must confront the outlined challenges head-on by developing generalizable algorithms, integrating domain knowledge, and validating systems in realistic settings. The coming years should focus on building a foundation of shared resources and on leveraging advanced AI paradigms to push the boundaries. With these continued efforts, the field is poised to usher in a new generation of intelligent OFS networks that are robust, reliable, and ready for deployment in diverse real-world applications. By systematically addressing the current bottlenecks and following the proposed roadmap, AI-enhanced optical fiber sensing can evolve from promising research into a transformative sensing technology across industries.

## Figures and Tables

**Table 1:** Summary of AI Algorithm Categories for Optical Fiber Sensing Applications.

AI Category	Representative Algorithms	Target Tasks Examples	Strengths	Limitations
Supervised Learning	SVM LR GPR DT/RF	-Event classification Strain/temperature estimation -Multi-parameter compensation	-High accuracy with labeled data -Interpretable	-Requires labeled data -May overfit on small datasets (DT)
Unsupervised Learning	K-means, Hierarchical clustering PCA AEs GANs	-Anomaly detection Signal clustering -Dimensionality reduction for visualization	-No labeling needed -Captures unknown patterns/outliers	-Results may be harder to interpret -Requires post-analysis
Reinforcement Learning	Q-learning DQN Policy Gradient Methods	-Adaptive sensor configuration -Resource-constrained data acquisition	-Suitable for real-time adaptation and system-level optimization	-Requires extensive training -Simulation or live environment needed
Deep Learning	MLP CNN RNN/LSTM	-Signal denoising -Spectral feature extraction -Vibration/acoustic classification -Field reconstruction	-Handles raw/high-dimensional data well -Learns features automatically	-Data hungry -Black-box behavior -Requires significant computation

**Table 2:** Summary of representative studies on AI techniques for OFS system optimization.

Study (Year)	Sensor Type	AI Technique	Improvement/Result
Feng et al., 2022 [47]	MMF speckle-based fiber spectrometer (strain/temperature)	CNN mapping speckle to spectrum	Achieved 0.1 pm spectral resolution; high SNR reconstruction of thousands of spectral lines.
Nguyen et al., 2021 [48]	MMF interference sensor (temperature) under heavy noise	MLP regression	Near-100% accuracy in retrieving temperature despite strong noise
Lalam et al., 2024 [49]	Distributed Brillouin sensor (BOTDA) over 25 km	DNN denoising + estimation	Reduced Brillouin frequency shift uncertainty from ~3 MHz to ~1.2 MHz; improved strain/temp accuracy by ~3× via noise suppression.
Yang et al., 2023 [63]	DAS seismic sensing (earthquake signals)	CNN denoising (DAS-Noise2Noise)	Recovered weak seismic signals buried in noise; outperformed band-pass filters in SNR, enabling detection of micro-seismic events.
Sun et al., 2007 [53]	LPFG pair sensor—bending curvature & temperature	FNN	Decoupled dual-parameter measurement with RMS error 0.0072 m^−1^ (curvature) and 0.19 °C, eliminating cross-sensitivity.
Zhu et al., 2022 [55]	Heterostructure fiber interferometer—bending vector sensor	Shallow ANN (1 hidden layer)	Predicted bending magnitude and direction from raw spectrum; 94% of predictions < 0.07 m^−1^ error
Karapanagiotis et al., 2023 [56]	BOFDA distributed sensor—strain & temperature crosstalk	GPR	Simultaneously output strain and temperature with ~45 µε and 2 °C error; effectively eliminated cross-talk between the two measurands.
Alshaikhli et al., 2024 [64]	FBG sensor—humidity & temperature (multi-param)	ANN with Levenberg–Marquardt training	Enhanced sensitivity by ~20 pm/%RH for humidity while accurately measuring temperature; improved linearity (R^2^ ≈ 0.95) over standard calibration.
Zhu et al., 2023 [57]	Vernier dual-interferometer (displacement)	Custom ML regression model	Real-time direct demodulation of measurand from interferogram; eliminated need for fringe unwrapping, greatly increasing readout speed.
Xue et al., 2023 [58]	Michelson fiber interferometer (chemical sensing)	LSTM	Achieved 97.5% concentration prediction accuracy; avoided errors from peak tracking and allowed lower sampling rate without loss of accuracy.
Liu et al., 2025 [65]	Overlapping FBG spectra (dense sensor network)	Bi-directional LSTM (Bi-LSTM)	Accurately demodulated overlapping FBG signals in real-time; enabled high-density FBG networks with fast, automated wavelength readout.
Wang et al., 2023 [60]	Optical fiber ballistocardiography (pulse monitoring)	LSTM regression	Compressed continuous heartbeat signal into HR/RR; R^2^ = 0.9946 for heart/respiratory rate, transmitting only vital signs instead of full waveform.
Peng et al., 2020 [61]	φ-OTDR distributed sensor (pipeline monitoring)	Deep neural classifier + unsupervised learning	Real-time event detection (intrusions ~85% accuracy, corrosion ~94%); streamed discrete alarms instead of raw data, achieving huge data reduction.
Wang et al., 2024 [62]	DAS seismic sensing (large data volume)	Recurrent autoencoder (RAE) with LSTM	Reached 512:1 compression ratio with minimal loss; reconstructed signal SNR ~40–44 dB at 8× compression

## Data Availability

No new data were created or analyzed in this study.
